# Supplementary material for: Copper Modulates Adult Neurogenesis in Brain Subventricular Zone
Source: Int J Mol Sci. 2022 Aug 31;23(17):9888. doi: 10.3390/ijms23179888 (PMC9456150; doi:10.3390/ijms23179888)
Supplement: Supplementary file 1 [file ijms-23-09888-s001.zip › ijms-1882325-supplementary.pdf]

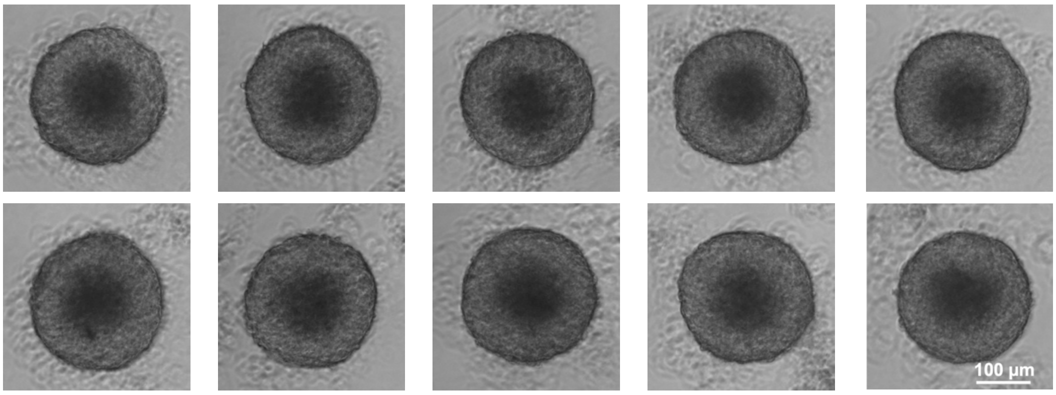

Supplementary Figure S1. The secondary neurospheres generated by this protocol average 216.8 ± 5.4 µm (RSD% 2.5, n =10) in the diameter.

Target	IF/IHC Dilutions	Host Species	Source and Identifier
**DCX**	1:1000	Rabbit	Abcam ab18723
**NeuN**	1:200	Rabbit	Abcam ab177487
**Ki67**	1:300	Rabbit	Abcam ab15580
**BrdU**	1:1000	Chicken	Abcam ab92837
**GFAP**	1:500	Rat	Invitrogen 13-0300
**Nestin**	1:200	Chicken	Novus NB100-1604
**Nestin**	1:200	Rat	Abcam ab81462
**CD133**	1:200	Rat	Invitrogen 14-1331-82
**CTR1**	1:200	Rabbit	Novus NB100-402
**MT3**	1:200	Rabbit	Proteintech 12179-1-AP

Supplementary Table S1. Primary antibodies and dilutions used for IF and IHC.

Genes	Genbank Accession	Forward primer (5'-3')	Reverse primer (5'-3')
**Ctr1**	NM_175090.4	CTTTACTGCGGTCGTGGGAC	GCCGCCTTAGTCTTCCTCTC
**Dmt1**	NM_001146161.1	AAAAGATGCCAGACGATGGCG	TGCTGTAGGCAGGGTTGATG
**Atp7a**	NM_001109757.2	GACTCTTCTGTGTGTGCGAGC	GAGGGGGTTAGCATTGTGGA
**Atp7b**	NM_007511.2	AGGAAGAACTTGGCGTCTGT	TGACTGCCTCTTGGTTGCTT
**Mt1**	NM_013602.3	AAGCGTCACCACGACTTCAA	AGGAGCAGCAGCTCTTCTTG
**Mt2**	NM_008630.2	CTTGAGCCAGAAAAAGGGCG	GAGCGTGATGGAGAGAAGCA
**Mt3**	NM_013603.2	CGGGAGGAACCAAGCTACG	TGCAGGAACCACCAGTAGGA
**b-catenin**	NM_007614.3	GGCGGCCGCGAGGTA	TTAGTGGGATGAGCAGCGTC
**Notch1**	NM_008714.3	CTTGCCAGGTTTTGCTGGAC	CTTTGCCGTTGACAGGGTTG
**Shh**	NM_009170.3	CCAACTCCGATGTGTTCCGT	TGTCGGGGTTGTAATTGGGG
**Dlx2**	NM_010054.2	CAGTTCGTCTCCGGTCAACA	ACTTCCAATACCGCTGGGTG
**Slit1**	NM_015748.3	CTGGAAGTGTGCTTTTCGATTG	GTTGTTGCCGTTGAGTTCCAG
**Slit2**	NM_001291227.2	TCGCTGGGGTTAGTGTTGTC	ATCACTGCAGACAAACTCTCGT
**Robo1**	NM_019413.2	CCCACCCCGACGATAGAATG	TTACAACGAAATGTGGCGGC
**Robo2**	NM_001358490.1	GCAATACCCCACCTCCACAA	TGTCCCCTCTCAGTTACTCTGT
**β-actin**	NM_007393.5	CCACCATGTACCCAGGCATT	CGGACTCATCGTACTCCTGC

Supplementary Table S2. Primers for mRNAs of research interest. Note: β-actin was used a housekeeping gene to calculate the relative expressions of genes of interest across samples.
